# Study of tribological properties of human buccal epithelium cell membranes using probe microscopy

**DOI:** 10.1038/s41598-022-14807-5

**Published:** 2022-07-04

**Authors:** N. A. Torhov, A. A. Mosunov, V. A. Novikov, I. V. Ivonin

**Affiliations:** 1 Sevastopol State University, St. University, 33, Sevastopol, Russia; 2 Tomsk State University, Av. Lenina, 36, Tomsk, Russia

**Keywords:** Biophysics, Biotechnology, Cell biology, Biomarkers

## Abstract

In this work demostrates a unique method for determining the absolute value of the friction force of a nanoobject on the surface of a cell membrane using atomic force microscopy. The tribological properties of membranes of adult human buccal epithelium cells in the presence of a protective adsorption buffer layer of ~ 100 nm on their surface were studied using atomic force microscopy in the contact scanning mode. Local mapping of the tribological characteristics of the surface was carried out, viz. friction *F*_*L*_ = *F*_*L*_*(x, y)* and adhesion *F*_*adh*_ = *F*_*adh*_*(x, y)* forces were measured. Studies of the friction force *F*_*fr*_ on the membrane surface at the nanolevel showed that its value varies discretely with an interval equal to *l*_*LF*_ ≈ 100 nm. It was shown that such discreteness is determined by the interval *l*_*LF*_ of the action of adhesive forces *F*_*adh*_ and indicates the fractal nature of the functional dependence of the friction force on the coordinate *F*_*fr*_ = *F*_*fr*_*(x)*. Thus, for nano-objects with dimensions ≤ *l*_*LF*_, the absolute value of *F*_*fr*_ decreases according to a power law with an increase in the size of the object, which contradicts the similar dependence of the friction force for macro-objects in the global approximation.

## Introduction

The intensive development of biomechanical systems implies integration of a person with various bioelectric and biomechanical devices. This requires development of methods for studying the biomechanical properties of the human body at the cellular level. In particular, the mechanical and tribological properties of the cell membrane determine the possibilities of its mechanical fixation and movement in the medium or along the external surface, as well as the ability of foreign objects [for example, zero-dimensional ones-quantum dots (molecules, fullerenes, etc.), one-dimensional-quantum threads (complex organic molecules, quantum wires), two-dimensional and three-dimensional-flat and three-dimensional nano-objects] attach and move along the surface of the cell itself^1^. The study of such properties of cells is the basis for atomic and molecular nanoengineering (manipulation of nanoobjects, creation of micro- and nanostructured devices) both on the surface and inside the cell. Most of the papers in this field were devoted to the interface of engineering, biology, biophysics, medicine (i.e. the mechanobiology), and are associated with the study of the reaction of cells and their components (membranes, organelles, complex organic molecules) to external and internal mechanical stimuli (signals).

The non-neoplastic epithelium organized in several layers and extending to the nasal cavity, mouth and oropharynx, conjunctiva, mucous-associated lymphoid tissue (MALT) (which is part of the autonomous mucosal immune system of a person), is more and more considered as a cellular and tissue material for non-invasive diagnostics of the state of human body^2,3^.

One of the methods for studying the mucosal epithelium cells of the oral cavity (the buccal epithelium of the mucous membranes) is the cytological method, which is based on the high sensitivity of the buccal epithelium cells to the state of human health. The good accessibility and non-traumatic reproduction of the collection of such cells relative to the oral mucosa as well as the simplicity and low cost of sample preparation make them a convenient biological material for in vivo diagnostics of most socially significant diseases^4,5^. These cells are highly informative regarding the influence of various physical, mechanical, chemical, environmental factors and drug effects on the human body^6,7^. The surfaces of the oral mucosa play a significant role in the sensory formation of the touch, smell and taste which is largely reflected in the psychoemotional state of a person^8^. All this makes the cells of the buccal epithelium one of the most convenient biological objects for obtaining important diagnostic and prognostic information about the state of human health, stress effects, the influence of environmental factors and xenogeneic intoxication, and pharmacology. It should be noted that these cells exist in a very aggressive environment in terms of temperature, humidity, pH and mechanical stress, which means that they can have a wide range of unique properties.

One of the most convenient tools for cell biology, providing the study of living eukaryotic cells with submicron and nanometer resolution, include methods of probe microscopy. In contrast to electron microscopy, probe research methods, in particular, atomic force microscopy (AFM) enable carrying out complex qualitative and quantitative studies of electro-physical, mechanical and, tribological characteristics of living biological objects under normal conditions (normal atmospheric pressure and room temperature) both in air^8^ and in liquid media^9^ given the proper tuning in a wide range of spatial and functional resolutions.

**Figure 1:**
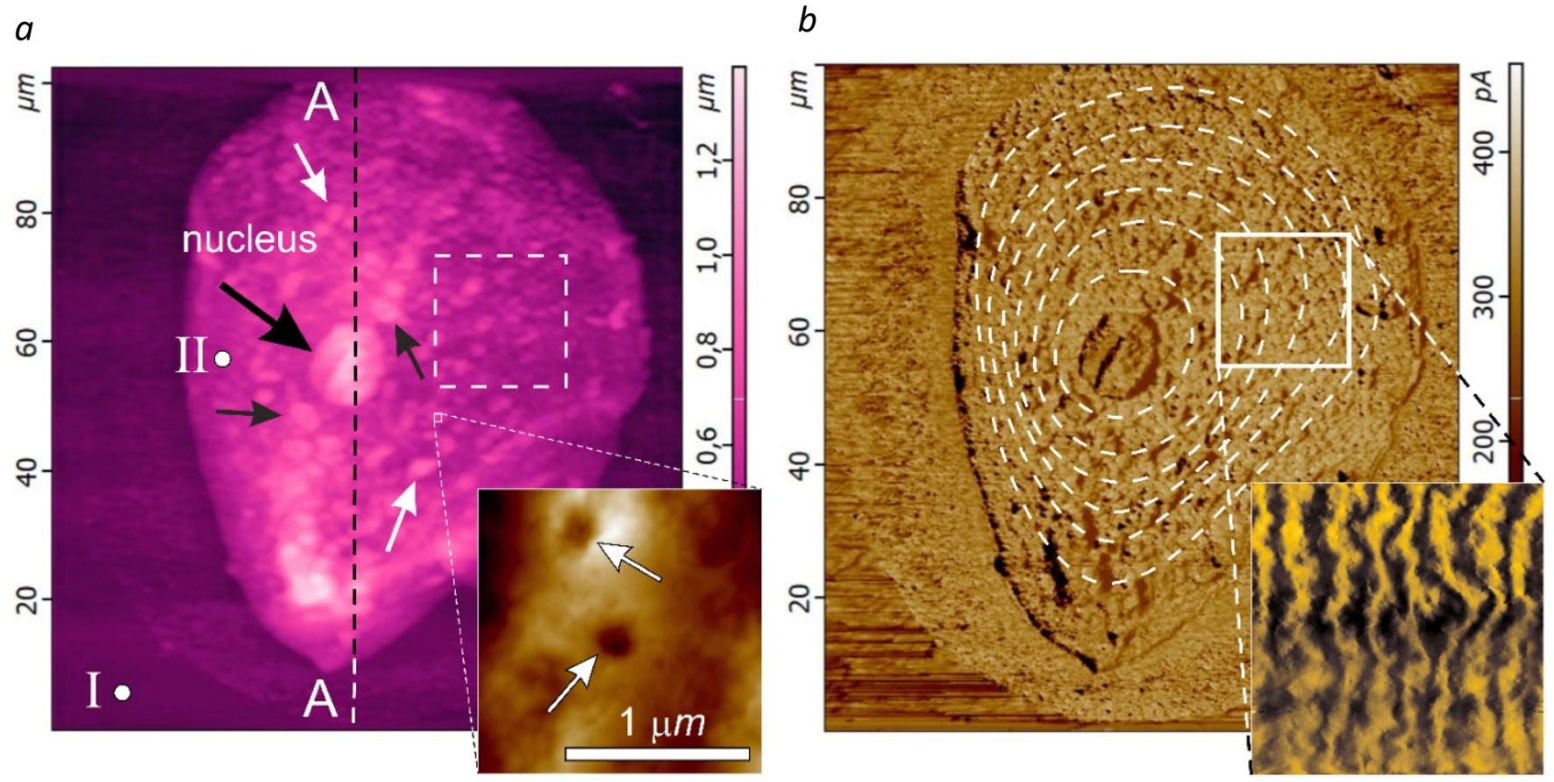
100 × 100 μm raster images of the surface area of n^+^-epitaxial Si {111} with an adult living cell of human buccal epithelium located on it, obtained by contact AFM scanning in constant force mode: relief h(x, y) (the black arrow indicates the location of the nucleus, white arrows point to some organelles and micronuclei inside the cell, the inset shows the area with nanopores) (**a**), and distribution of lateral forces LF(x, y) (dotted line denotes the preferred orientation of the contrast of lateral forces, the inset demonstrates raster LF image of the selected 10 × 10 μm region of the cell surface area) (**b**).

The membrane of a living cell due to its selective permeability to substances, ensures not only its integrity, but also maintains its homeostasis (internal composition). At the same time the cell membrane features not only the barrier, but also the transport functions. In the scientific literature the transverse transport of substances through the cell membrane is well described^10–12^. A distinction is made between the passive concentration-gradient induced diffusion transport through the membrane (for fat-soluble substances)^13–15^, and the transport against the gradient, which is possible only by a certain carrier with the expenditure of energy. For example, there are carriers that bind to the transported molecules and move with them through the membrane (such as glucose)^16,17^. There are stationary carriers that form a pore in the membrane, i.e. a transfer channel (for example, Fig. 1a, inset)^18–20^. The transfer in the channel can be initiated due to the conformation of the protein (for example, the sodium–potassium pump). The transport functions of larger nano-objects across the cell membrane can be realized through the mechanisms of endocytosis and phagocytosis^21–23^.

At the same time the transport functions of the membrane of a living cell along its surface are practically not studied at present. Meanwhile, they play a fairly significant role in almost all of its functional characteristics. It is clear that in order to bind with a mobile carrier, or interact with a transport channel, an external object (for example, a micro- or nano-particle) must move to them from the point of attaching to the surface, while doing some work A = *l*_*f*_*F*_*fr*_ to overcome the friction forces *F*_*fr*_ = *F*_*L*_ = *F*_*adh*_.

Despite the obvious progress in this field the amount of experimental data on the tribological properties of the membranes of human buccal epithelium cells is still extremely insufficient. In some cases this is due to the use of insufficient resolution, in others the lack of modern geometric methods and AFM techniques that allow recording a full range of Brownian, micromechanical, and tribological surface parameters in a single measurement cycle.

The aim of this work is to study the complex functional tribological properties (friction forces *F*_*fr*_ = *F*_*fr*_(*x*,*y*) and adhesion *F*_*adh*_ = *F*_*adh*_(*x*,*y*)) of the shell of living cells of the human buccal epithelium (hereinafter referred to as the cell).

## Materials and methods

### Object of study

The object of investigation was the living cells of the human buccal epithelium obtained by the method of liquid cytology. This method included the following steps: mechanical sampling (scraping) from the inner surface of the cheek of the oral mucosa, washing the scraping in phosphate buffer (3.03 mM phosphate buffer with the addition of 2.89 mM calcium chloride of 5 ml in volume, pH 7.0), placing this mixture in a test tube and separation of its contents in a centrifuge for 5 min at 1700×*g* (5000 min^−1^), taking of an aliquot of a buffer solution containing a suspension of live cells of the buccal epithelium from a centrifuge tube. An epitaxial structure of silicon with p-type conductivity p–p + − Si {111} with an irregularity size < 20 nm was used as a substrate material. To increase the hydrophilic properties and increase the surface adhesion, the silicon structure was treated in hexamethyldisilazane vapor.

After placing a drop of an aliquot on the epitaxial surface of silicon Si {111} it was dried in air at normal atmospheric pressure and temperature T ≤ 40 °C. The process of removing moisture from the aliquot was controlled visually and the time usually did not exceed 10 min. Living cells in an aliquot naturally settled on the epitaxial silicon surface and remained in such state for 2–2.5 h after the evaporation of the bulk of moisture. This was indicated by the irreversible changes in the state of the surface and morphology of their membranes that occurred according to the studies after 3–4 h of exposure of the cells to air. Note that the used drying regime left an adsorption layer of a buffer solution on the surface of the cells, which maintained their viability during a relatively long exposure to air at normal conditions (NC). This layer prevented the cells from drying out and maintained their viability for > 2.5 h of exposure to air. This fact was confirmed by the dye exclusion method (trypan blue). After 2.5 h, most of the cells were resistant to the dye.

### AFM methods

Investigations of the geometry of the relief (Fig. 1a) of the surface of cell membranes and their tribological properties (Fig. 1b) were carried out in air under normal conditions using an atomic force microscope (AFM) NTEGRA-SPECTRA in a contact (constant forces F_Z_ = F_const_) and hybrid scanning modes in the Resource Center ‘Molecular structure of matter’ of the Sevastopol State University.

**Figure 2:**
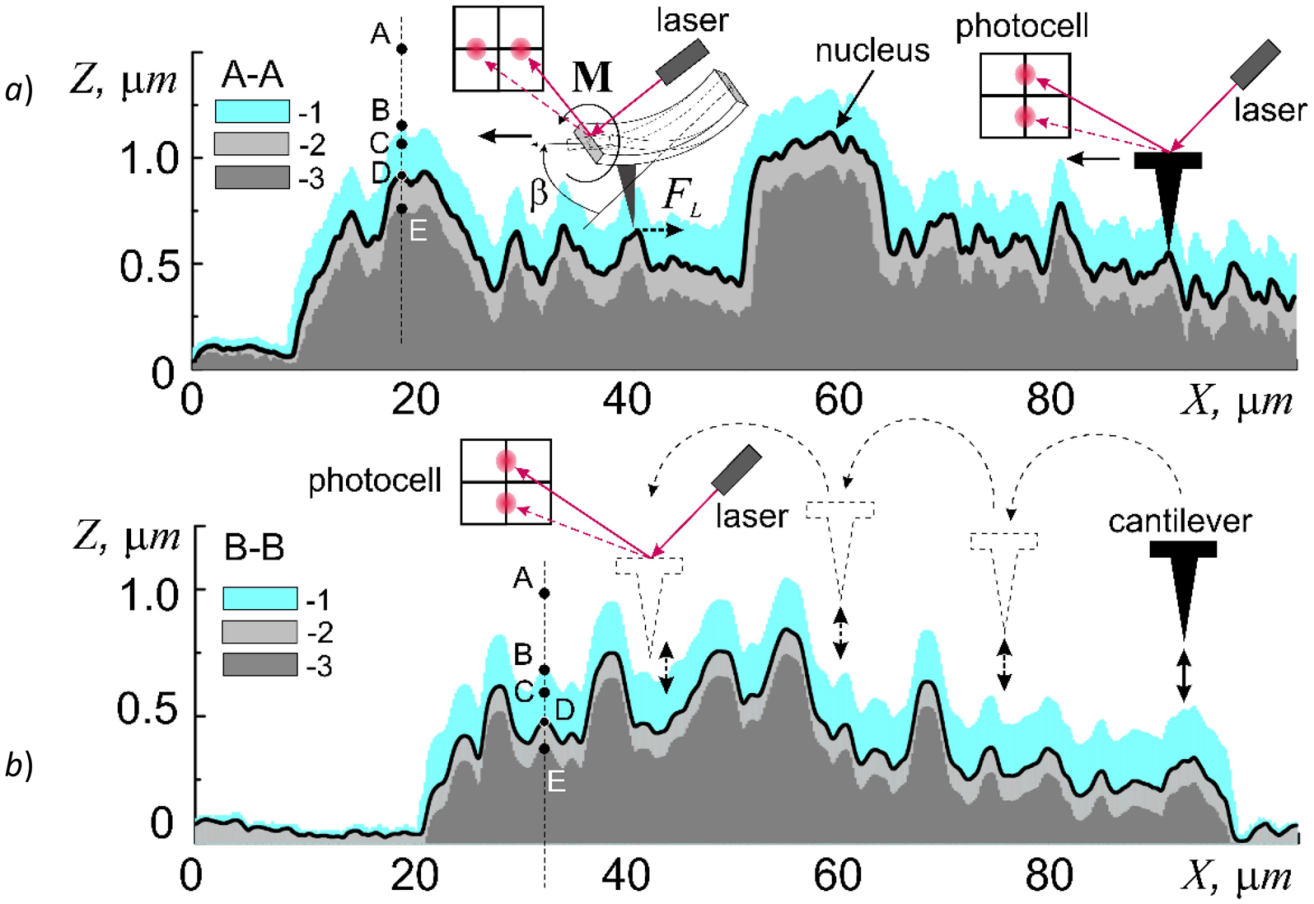
Profiles of cross-sections A–A and B–B shown in Fig. 1a of a buccal cell of human epithelium (1—adsorption layer, 2—elastic area, and 3—plastic deformation area). Schematic representations of the principles of AFM operation: contact (**a**), and hybrid (**b**) scanning modes.

In the contact scanning mode (Fig. 2a) the constant mechanical contact of the tip of the cantilever (the probe) with the surface is maintained at a constant pressing force F_const_. An important feature of this method is the possibility of a direct control of the static values of F_const_ ≥ 0. In addition, this scanning mode allows recording the LF signal (Fig. 1b), which is responsible for the torque of the cantilever beam around the longitudinal axis, which characterizes the magnitude of the lateral force F_L_ (Fig. 2a, **M**). When the cantilever moves longitudinally under the action of lateral (in the (x, y) plane) forces on the probe F_L_ = F_fr_ (the friction forces), a torque **M** arises deflecting the laser beam reflected from the outer side of the cantilever beam not vertically, but horizontally. It is clear that at a constant pressing force F_const_, the friction force F_fr_, and, consequently, the torque **M**, will completely depend on the nature of the underlying surface, in particular, the phase composition of the cell membrane surface, which determines the physical mechanisms of the adhesion of the cantilever tip to the surface. To construct raster LF-images of AFM during the surface cartography as well as for numerical processing of the results, the ‘Dynamic’ mode was used, in this mode the mean values of the LF-signal amplitude over the interval passed between two points were recorded. The contrast of lateral forces arising in this way on the raster image indicates the difference in tribological properties of different areas of the cell membrane surface. For more accurate quantitative analysis (for example, when analyzing linear profiles of cross sections), the ‘Stable’ mode was used, in which the value of the LF signal amplitude was fixed directly at each point.

The hybrid scanning method is currently one of the most advanced probe methods for studying surfaces, because it combines the advantages of contact and semicontact modes (Fig. 2b). This combination allows in a single measurement process to simultaneously study the surface relief and a set of its tribological characteristics, such as lateral forces F_L_ = F_L_(x, y) − friction forces F_fr_, and adhesion F_adh_ = F_adh_(x, y). Briefly, the essence of this method lies in the fact that during the scanning with a step-by-step movement of the cantilever at each point after the initial force F_const_ = F_Z_(Z = 0) is applied, the approach F_Z_ = F_Z_(Z↓), and the retraction F_Z_ = F_Z_(Z↑), curves are measured (Fig. 2b). The values of the mechanical characteristics are obtained by the mathematical processing of the linear sections of the force curves in the vicinity of the point z_0_.

As a measuring probe we used an HA-FM/W_2_C cantilever, which is a micromechanical silicon device consisting of a rectangular 3.6 × 1.6 mm silicon base with a thickness of 0.4 mm in the center of the smaller side of the upper face of which a beam 183 μm long, 34 μm wide and 3 μm thick was formed. The top surface of the beam was covered with a reflective gold plating. On the lower side of the free end of the beam a tip with a height of 12 μm, apex angle *α* ≈ 22° and a radius of curvature r ≤ 6 nm (hereinafter referred to as the probe) was formed, covered with a high-strength layer of tungsten carbide W_2_C. The design parameters of the needle and the conditions for carrying out probe measurements (low hardness of the studied biological objects) imposed restrictions on the choice of models of contact interaction of the tip of the needle (probe) with the surface. Taking this into account we used the conical DMT model (the model of Derjagin, Muller and Toropov) used to calculate the values of micromechanical parameters in cases when the dimensions of the elastic deformation of the surface significantly exceed the radius of curvature of the tip of the needle, r^24^.

Immediately before carrying out quantitative measurements of the surface parameters, the elasticity coefficient of the cantilever beam, K ≈ 5.6 N/m, was calibrated at its resonance frequency f = 107 kHz, taking into account the spectrum of thermal oscillations. The cantilever was approached to the surface in the ‘touch’ mode, which provided a fairly accurate step-by-step approach of the probe to the surface.

The choice of the scanning parameter ‘setpoint’ (the pressing force F_const_), was carried out by the method of force spectroscopy F_Z_ = F_Z_(Z) (the force curves), by taking the approach and retraction curves. These curves describe the dependence of the bending amplitude of the cantilever beam on the pressing force F_z_ (the degree of extension of the scanner piezotube along the Z-axis).

### Friction force

Continuum mechanics makes it possible to describe the frictional interaction (friction force F_fr_) of two macroscopic bodies: static friction, sliding friction, rolling friction and spinning friction. Since the frictional interaction of the probe with the surface in the conducted experiments was carried out at low loads in the region of elastic stresses, the torsion angle of the cantilever beam is small, and instead of the spinning friction model in the first approximation one can use the sliding friction model. In this case both the deformation and molecular components are present in the mechanisms of frictional interaction (dry, boundary, mixed and viscous)^25^. In the case when the pressing force F_⊥_ (transverse component) leads to plastic deformation Δ*h*_*stif*_ or destruction of the material, it plays the main role in the formation of F_fr_^26^. The elastic deformation component, for obvious reasons, will not contribute to the friction force.

In the presence of only the deformation component the Amonton-Coulomb law describes the linear dependence of the friction force F_fr_ on the normal load F_⊥_:

1 $${F}_{fr}=\mu {F}_{\perp }$$

The friction coefficient *μ* is a dimensionless quantity and is usually *μ* << 1. In the AFM method without taking into account the weight of the nanoparticle *F*_⊥_ = *F*_*const*_. Additional consideration of the molecular component, i.e. the adhesion force F_adh_ leads to the appearance of the second term in the Amonton–Coulomb law:

2 $${F}_{fr}=\mu {{F}_{\perp }+F}_{adh}$$

The adhesion forces F_adh_ change nonlinearly depending on the coordinate F_adh_ = F_adh_(Z) and according to the Lennard–Jones potential U_LD_ (3)^27^ are characterized by strong repulsion at close distances and weak repulsion at large ones:

3 $${U}_{LD}\left(\mathrm{r}\right)={U}_{0}\left\{-2{\left(\frac{{\mathrm{r}}_{0}}{\mathrm{r}}\right)}^{6}+{\left(\frac{{\mathrm{r}}_{0}}{\mathrm{r}}\right)}^{12}\right\}$$

Here the vector **r**_o_ is the equilibrium distance between atoms and U_0_ is the minimum value of the potential energy in the equilibrium system–the bottom of the potential well. The first term in this expression describes mainly the dipole–dipole attraction of atoms and the second much shorter-ranged-their repulsion at small distances. The radius of action of Van-der-Waals forces (tens of nanometers) significantly exceeds the radius of wave functions overlapping for interacting nano-objects (units of nanometers). Van der Waals forces arise due to the fluctuation nature of the orientational electromagnetic forces of interaction between bodies, which are obtained as a result of averaging over the equilibrium distribution of the orientations of interacting dipoles, induction interaction (usually observed at elevated temperatures) and dispersive quantum mechanical interaction. We also note that the competition between the Van-der-Waals forces of attraction and repulsion often takes an active part in the coagulation of colloidal systems, which in many cases include cell membranes and which we will further demonstrate in the experiment for non-living cells.

From Eq. (2) one can obtain the main condition for the presence of adhesive forces in the frictional interaction:

4 $$1<\mu =({F}_{fr}+{F}_{adh})/{F}_{\perp }$$

When studying the tribological properties of cell membranes we immediately come across a number of features.

First, the physical mechanisms of friction at the micro- and nanoscale levels may differ significantly from the similar mechanisms of frictional interaction of macro-objects. The effective contact boundary (molecular points of contact during intermolecular interaction) turns out to be significantly curved^25^. In addition, in the local approximation, with a decrease in the linear scale, the multifractal shape of the surface begins to play a significant role, as the surface exhibits several levels of roughness with their characteristic dimensions and filling density, depending on the size of the frictional contact^25^. These circumstances significantly complicate the task of determining the real contact surface of the contacting bodies and calculating the friction force. In addition, with the decrease in the scale of objects due to their low mass (*F*_⊥_ << 1), the adhesion forces begin to noticeably prevail over the deformation component^25^.

Second, the medium of the cell membrane (peptidoglycol gel^28^) is highly heterogeneous in composition and shape and has the properties of two-dimensional objects in the presence of boundaries. In this case the capabilities of continuum mechanics may be insufficient.

Third, the force required to move a nano-object over a surface strongly depends not only on its nature, but also on its state at a given time, for example, on the features of its preliminary preparation and the presence of an adsorbate^26^.

And, fourth, the cell membrane is a strongly nonequilibrium thermodynamic object with negative entropy.

Nevertheless, despite the seriousness of all these considerations, the authors of^29^ report that at the nanoscale level most of the basic laws of frictional interaction in the framework of continuum mechanics can be applied. So, according to^29^, as the adhesion between the contacting surfaces at the nanoscale level decreases, a transition occurs from nonlinear (2) to a linear dependence of the friction force F_fr_ on the load F_⊥_ (1). In this case, the friction force linearly depends on the number of interacting atoms in the contact, which in general is analogous to the area.

Unfortunately, at present, there are no AFM methods for direct measurement of the friction force F_fr_ at the nanoscale level. This is due to the fact that when recalculating it into the value of the force parameter of the received LF signal, it is necessary to simultaneously take into account not only the bending of the beam at an angle β, but also consider its torque around the longitudinal axis **M** (Fig. 2a, **M**). While well-mastered ‘bending’ methods are used to calibrate the elastic properties of the beam, there are currently no methods for calibrating the torque of the cantilever beam in the presence of bending. In this regard, in the comparative analysis of friction forces, we will use relative values: amperes [LF] = A, or ‘arbidden units’.

### Method of fractal surface analysis

The analysis of the relief and functional characteristics of the surface of the cell membrane was carried out within the framework of the geometry of fractional dimensions using mathematical methods of fractal geometry.

In the general case the task of determining the measure M of a biological object is to determine how many times N a measured object embedded in a limited space R^D^ can be filled with a certain measuring (calibration) object described by the function $l(\delta ) = \gamma (D_{{\text{T}}}^{{}} )\delta_{{}}^{{{D_{M}{}} }}$, where *γ* is the normalization factor, *δ* is the dimensionless scale, and D_M_ is the Minkowski dimension (the dimension of a bounded set in a metric space). Then, according to^30^, $N$ = $1/{\delta }^{{D}_{M}}$. Since in this paper we deal only with subsets of metric spaces and functional subspaces defined on them, instead of *D*_*M*_ we operate with the concept of Hausdorff-Besicovich dimension *D*_*H*_ (hereinafter the Hausdorff dimension or Hausdorff dimension), close to the concept of Minkowski dimension *D*_*H*_ ≈ *D*_*M*_^30^. Thus, the dimension *D*_*H*_ of a bounded set in a metric space can be represented as

5 $${D}_{H}={D}_{M}=\underset{l\to 0}{\mathrm{lim}}\frac{\mathrm{ln}(\eta )}{\mathrm{ln}(\varsigma )}$$

where *η* is the minimum number *N* of the sets of linear size *l* with which it is possible to cover (fill) the measured set when the diameter *l* is decreases by a factor of *ζ*^30^.

Then the measure *M* of a biological object can be written as:

6 $$M=N\left(\delta \right)l(\delta )=\gamma (D){\delta }^{{D}_{T}-{D}_{H}}$$

where *δ* = 1/*ζ* and *D*_T_ = 1, 2, 3 is an integer topological dimension.

In the case of self-similar (for example, fractal) sets, the Hausdorff dimension can be considered as the similarity dimension *D*_*H*_ = *D*_*S*_ or the fractal dimension *D*_*f*_.

If in Eq. (6) as the normalization factor γ(*D*) we take the known value of the measure *M*_0_ of some investigated biological object, then Eq. (6) can be represented as:

7 $$M={M}_{0}{\delta }^{{D}_{T}-{D}_{f}}$$

From here, by taking the logarithm of the right and left sides, we obtain an expression for the dimension *D*_*f*_ expressed in terms of the measure *M* of the object:

8 $${D}_{f}=\frac{\mathrm{ln}\left[\frac{{M}_{0}}{M}\right]}{\mathrm{ln}\delta }+{D}_{T}$$

Recall that an ideal fractal object is formed by nested disjoint sets of self-similar figures and can be characterized by the following parameters: local approximation limit *L*, fractal (fractional) dimension *D*_*f*_, scaling coefficients *ζ* and *η*, the law of affine transformation x_i_ = **A**x_i−1_^30^. Recall that *D*_*f*_ = *D*_*T*_ for *l* ≥ *L*.

At the same time, most Brownian or chaotic objects in nature, which often include biological systems, are not self-similar in the literal sense. We can only talk about a certain statistical similarity and statistical self-affinity-similarity in a certain interval of measuring scales characterized by the average value of the fractal (fractional) dimension over the set. In this case, the object under study can be characterized not by one, but by several (depending on the measuring scale *l*) values of the fractal dimension *D*_*f*_ = *D*_*f*_(*l*)—the so-called multifractal objects^30^.

Carrying out simple transformations one can find that in the local approximation (*l* < *L*) the ratio of measures of fractal objects corresponding to different linear dimensions *d*, obeys a slower power-law dependence than in the global (*l* ≥ *L*) approximation:

9 $$\frac{{M}_{i}}{{M}_{i+1}}={\left[\frac{{l}_{i}}{{l}_{i+1}}\right]}^{{D}_{T}-{D}_{f}}$$

From Eq. (8) the value of *D*_*f*_ can be determined as:

10 $${D}_{f}=\frac{\mathrm{ln}\left[\frac{{M}_{0}}{{M}_{i}}\right]}{\mathrm{ln}\left[\frac{{l}_{i}}{{l}_{0}}\right]}+{D}_{T}$$

and also the measure of the object *M*_*i*+1_ of the next level of similarity:

11 $${M}_{i}={M}_{0}{\left[\frac{{l}_{i}}{{l}_{0}}\right]}^{{D}_{T}-{D}_{f}}$$

In practice, the triangulation method is often used to determine *D*_*f*_^30^. Calculation of the *D*_*f*_ value by the triangulation method consists of sequential approximations of the surface by a set of pyramids and measuring the area of their lateral surfaces. The accuracy of measuring the surface area *S* in this case will depend on the number of such pyramids (the number of partitions), which is determined by the value of the measuring scale *δ*.

### Ethics declarations

This study was performed in line with the principles of the Declaration of Helsinki. Approval for studies including collection of buccal epithelium was granted by the Ethics Committee of Sevastopol State University (Study No. 3; July, 15th 2021).

Buccal epithelium was collected in accordance with the code of conduct of research with human material in the Russian Federation. All subjects gave written informed consent.

## Results of experiment

### Force spectroscopy of the cell wall

To understand the peculiarities of probe measurements of cells at the submicron and nanoscale level and the choice of modes of force action when scanning the shell, let us consider the description of the spectra *F*_*Z*_ = *F*_*Z*_(Z) of the main significant areas of the reference approach and withdrawal curves describing the features of the mechanical interaction of the probe with the surface depending on the distance of the probe-surface Z.

**Figure 3:**
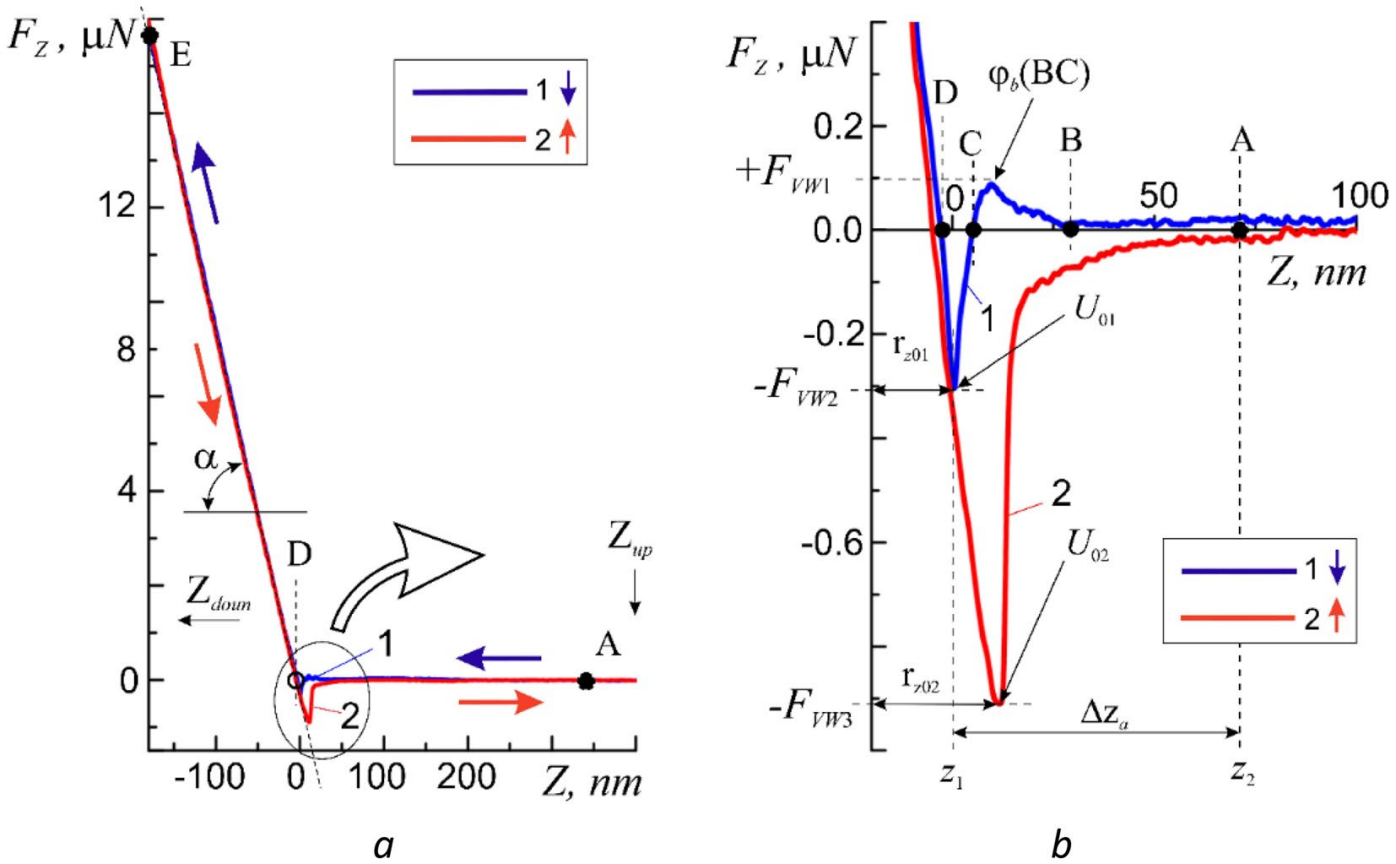
Approach 1 and withdrawal 2 curves (inset) obtained on the epitaxial silicon surface (**a**), and a more detailed image of the same curves in the region of the adsorption layer (**b**).

In the general case, before carrying out the force measurements of the AFM in the ‘setpoint’ parameter, the initial value of the force action of the probe *F*_*const*_ = *F*_*Z*_(*z*_0_ = 0) on the membrane at the point of its contact with the surface was set, i.e. the level of the initial force action corresponding to the zero coordinate along the Z-axis (*z* = 0). At the start of the measurement the initial force action was removed, the probe was retracted from the surface at a predetermined distance Z_*up*_ ≈ 500 nm, and the approach curve *F*_*Z*_ = *F*_*Z*_(Z↓) was measured by forcibly lowering the probe to the lower point Z_*doun*_ ≈ − 300 nm (Fig. 3a). After that the *F*_*Z*_ = *F*_*Z*_(Z↑) withdrawal curve was measured up to the upper point Z_*up*_* ≈ *500 nm. As a reference we used the spectra of the approach and withdrawal curves recorded on the free silicon surface with known mechanical characteristics (Fig. 1a, point I).

Let us consider the spectral characteristic of the approach process (Fig. 3a, curve 1). The horizontal section AB describes the process of the approach of the cantilever to the surface from a sufficiently large distance between them, i.e. in the absence of interaction *F*_*Z*_ = 0. As it approaches the surface the probe becomes influenced by Van-der-Waals forces (F_VW_). First, the repulsive forces (*F*_*Z*_ > 0) *F*_*Z*_ =  + *F*_*VW*1_(BC)* ≈ *0.1 μN begin to act due to a small potential barrier [*φ*_*b*_(BC)] = eV in the segment BC, and then act the forces of attraction (*F*_*Z*_ < 0) *F*_*Z*_ = − *F*_*VW*1_(CD)* ≈ *− 0.31 μ*N* of the potential well *U*_01_ (section CD), which reach their maximum value at the immediate vicinity of the surface (point r_z0_) (Fig. 3b, curve 1). The barrier *φ*_*b*_(BC) most likely describes the process of overcoming the surface tension forces of the adsorption layer by the cantilever. The depth [*U*_01_] = eV of the potential well determines the maximum force of attraction of the probe to the surface and the work of adhesive forces. With further movement of the cantilever towards the surface beyond the point D, the repulsive forces *F*_*H*_ > 0 (*H*—Hooke's law) begin to act on the probe preventing its penetration into the near-surface region (Fig. 3a, section DE). The linearity *F*_*Z*_ = *F*_*Z*_(DE) of the section DE of the approach curve indicates the presence of elastic forces leading to the appearance of reversible elastic deformations at the point of action of the probe on the surface described by the Hooke's law viz. the reaction force is directly proportional to the change in the linear size Δ*z*:

12 $${F}_{z}=K\times \Delta z$$

where [*K*] = N/m is the coefficient of elasticity. For this process *K* = 8.3 ± 0.2 N/m. Here it is convenient to introduce the concept of an effective surface, i.e. the *z*-coordinate (point D) at which repulsive forces arise.

The movement of cantilever towards the surface beyond the point E leads to a critical increase of the force *F*_*Z*_ > *F*_*H*_, violation of the linearity *F*_*Z*_ = *F*_*Z*_(DE) and the appearance of plastic deformations Δ*h*_*stif*_, which were not considered in this work. These areas are shown schematically on the A–A profile of the cross-section of the surface relief of the shell surface of the cell under study (Fig. 2).

The withdrawal curve (Fig. 3a, curve 2) in the general case has similar characteristic regions, which can differ significantly from the similar regions of the approach curve depending on the state of the surface. Obviously, for clean homogeneous solid surfaces the approach and withdrawal curves must coincide. The discrepancy between the approach and withdrawal curves (for example, a large by modulus force of attraction |− *F*_*VW*3_| > |− *F*_*VW*2_|, the absence of the barrier *φ*_*b*_, an increase in the coordinates of the bottom of the potential well *U*_02_), i.e. the hysteresis, usually indicates the presence of certain features in the state of the surface and near-surface area. The higher coordinate of the potential well r_*z*02_ > r_*z*01_ indicates the presence of an adsorption layer (adsorbate) with an effective thickness Δz_a_ < 100 nm on the surface at the point where the probe touches. The presence of a thin nanometer layer of adsorbate in this case is most likely caused by the remains of the buffer solution. According to the theory of capillary phenomena by T. Young, P. Laplace and J. Gibbs, the adsorption effect is largely determined by the Van-der-Waals forces^31^. The coincidence in the DE region of the linear sections of the approach and withdrawal curves as well as their identical slope (angle α) confirm the uniformity of the mechanical properties of the near-surface region of the epitaxial silicon laye and also indicate the correct tuning of the AFM system and the operation of the cantilever. Thus, for the Si surface the shape of the approach and withdrawal curves fully corresponds to the well-known physical model of the interaction of two atoms by means of Van-der-Waals forces and located at a distance **r** from each other in accordance with the Lennard–Jones potential U_LD_ Eq. (3).

Thus, it becomes clear that depending on the state of the surface and the value of the pressing force it is possible to exert various force impacts on the surface of the shell (approach curve) with the registration of the values of the parameters of the membrane response reactions (withdrawal curve) to an external stimulus. The correct choice of the initial value of the force *F*_*Z*_ = *F*_*const*_ during AFM measurements of the surface of a biological object is a very important preparatory stage. For example, an excessively large *F*_*const*_ value can lead to large plastic deformations or even rupture of the cell membrane and an excessively small value can lead to the excessive sensitivity of the method and the occurrence of various kinds of interference associated in most cases with various inhomogeneities.

According to the preliminary results the cell membrane possesses good elastic properties and withstands elastic deformations along the Z-axis significantly exceeding 300 nm with a force *F* > 28 nN. In addition, elastic deformations comparable with the thickness of the cell indicate the non-destructive action of the probe (integrity, tightness of the membrane), which excludes the leakage of the contents of the cell under study to the outside.

To select the optimal value of the constant pressing force *F*_*const*_ we selected relatively flat areas of its surface (Fig. 1a, point II) under which there were no organelles (Fig. 1a, arrows). As a result, for the AFM measurements by the contact method the pressing force *F*_*const*_* ≈ *18–22 nN was chosen providing not only a sufficient force effect, but also a good signal-to-noise ratio at a level of elastic deformations of at least 150 nm. Note that repeated scanning in more gentle modes (at *F*_*const*_ < 5 nN) did not reveal any destructive effects of the cantilever.

Preliminary studies by the AFM Kelvin probe method have shown the halo around the cell in Fig. 1 is due to the difference in electrostatic potentials Δφ of the cell and the silicon surface and is not associated with a violation of the integrity of the membrane. The electrostatic field surrounding the cell and caused by the potential difference Δφ stimulates the predominant precipitation from the buffer solution in the area of the halo during sample preparation when the aliquot is dried. The study of the electrical characteristics of the cell membrane requires more detailed independent studies and was not considered in this work.

**Figure 4:**
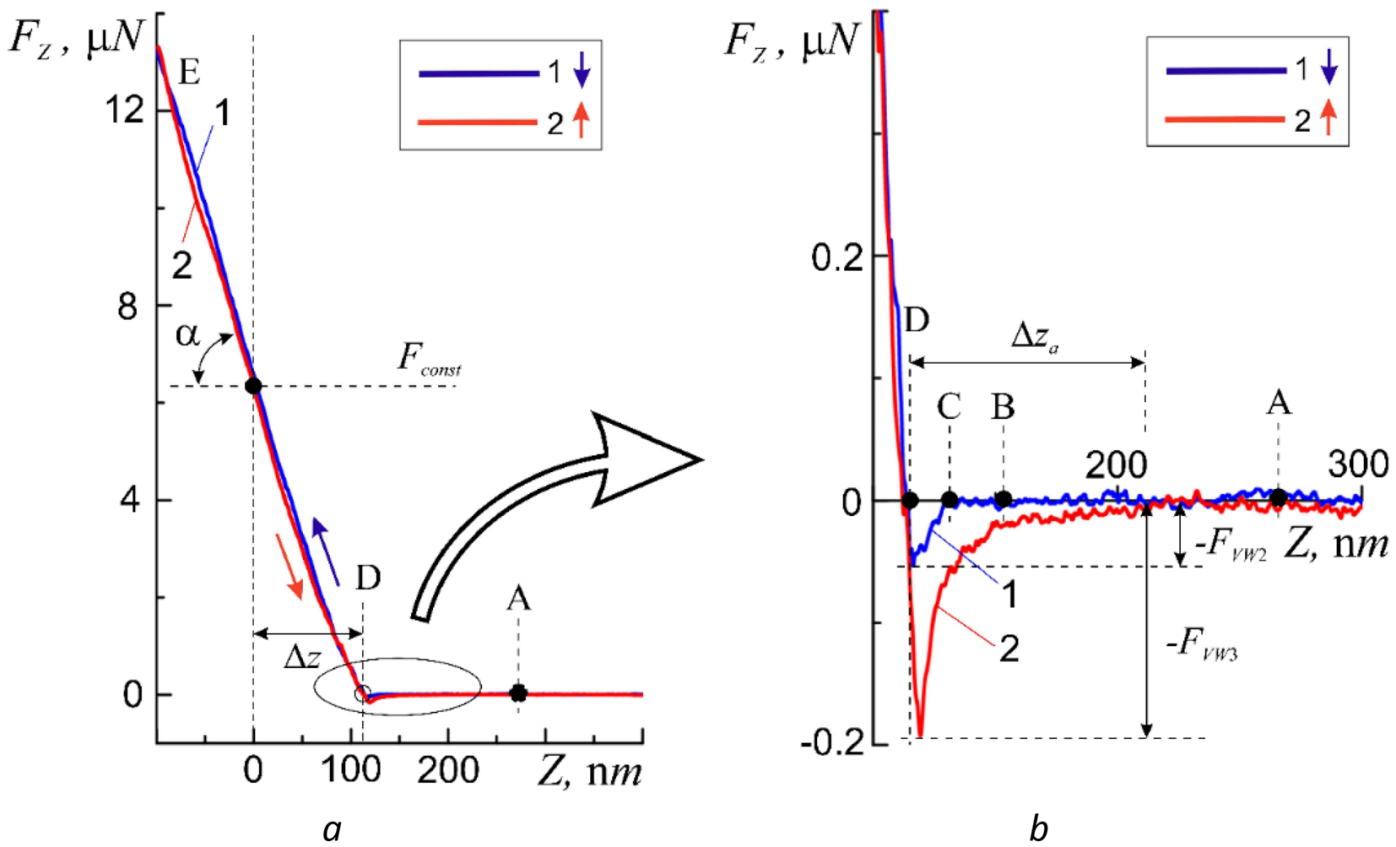
Approach 1 and withdrawal 2 curves obtained on the membrane surface of a human buccal epithelium cell at one point for the following values of the initial force action *F*_II*,const*1_ = 6.2 μN (**a**), and a more detailed image of the same curves in the adsorption layer region (**b**).

As it was expected the features of the micromechanical properties of the cell wall manifest themselves in significant differences in the approach and withdrawal curves (Fig. 4). It was found that the reaction of the membrane of a living cell to the action of the probe is not a simple mechanical process but, depending on the method of action, is a selective and non-trivial process and will be described in detail in subsequent publications. In this paper we were only interested in the elastic reaction of the shell.

The greater slope (angle α) of the linear sections of the approach curves indicates a greater elasticity of the cell membranes, i.e. a greater value of the elasticity coefficient *K*. According to Fig. 4a at *F*_*const*_ = 6.2 μN the deformation was Δh_dfrm_ = 115 nm and *K* = 67.4 ± 0.15 N/m. In this case the region of deformations Δ*h*_*dfrm*_ ≤ 115 nm falls in the elastic region. The regions of plastic deformation were not considered in this paper. In accord with the authors of^32^ the coefficient of elasticity of the cell membrane turned out to be higher than the coefficient of elasticity of the cantilever beam.

Differences are also visible in the near-surface region of action of adhesive forces. First of all attention is drawn to the absence of repulsive forces (barrier φ_b_(BC)) on the approach curves when approaching the surface and the significantly shallower depth of the ‘potential well *U*_0_’ *F*_*VW2*_ (Fig. 4a, curve 1). This fact indicates a relatively low hydrophilicity of the cell membrane. In other words micro- and nano-objects approaching this point in the absence of a noticeable effect (pressing force *F*_*const*_) on the membrane will be very weakly held by its surface. This circumstance, on the one hand, can allow the cell to move more freely in the external environment due to low wettability and, on the other hand, it is easy to overcome surface tension forces due to the lack of hydrophobicity.

In the general case, withdrawal curves have a more complex dependence on the coordinate and, most likely, cannot be characterized by elasticity coefficient. At the same time even a relatively small effect of an external nano-object on a cell leads to an increase in the adhesive forces of attraction, which allows the cell to fix itself on external objects or to retain on its surface the external micro- and nano-objects leaving it.

Withdrawal curves have more in common with the test withdrawal curves (Fig. 4). They also have a region of elastic deformations DE, a potential well region BD, and a neutral region AB of the absence of interaction. According to the behavior of the withdrawal curves a rather thick Δ*z*_*a*_ < 100 nm adsorption layer of the buffer solution was present on the cell membrane surface (Fig. 4b, Δ*z*_*a*_). This layer protected the cells from complete drying out for a sufficient (3–4 h) period of time needed for measurements. Such conditions for AFM scanning of the surface of a living cell are intermediate between measurements of a completely dried (dry) non-living cell and measurements of living cells in a liquid medium. Obviously, such a mixed scanning method for AFM measurements of biological objects is easier to use and to some extent combines the advantages of the classic ‘dry’ and ‘wet’ scanning methods.

The authors of^7,9^ observed a similar behavior of the approach and withdrawal curves, but provided no explanation when studying the elasticity of local 20 × 20 µm sections of the membranes of human intestinal cells Caco-2 by AFM force spectroscopy.

### Tribological properties of the cell membrane surface

Due to the fact that the weight of the observed micro- and nano-particles is insufficient for the occurrence of any noticeable plastic deformations of the shell Δ*h*_*stif*_ (for example, the weight of a lead ball with a diameter of 1 μm is only ~ 4 × 10^–13^ N), the first term in Eq. (2) can be excluded, then the condition Eq. (4) 1 << μ is satisfied and the friction force will be completely determined by the adhesion forces *F*_*adh*_≡*F*_*VDV*_ on the membrane surface, i.e. by the Van-der-Waals forces:

13 $${F}_{fr}={F}_{adh}$$

It is clear that for satisfying conditions (4) and (13) in AFM it is necessary that F_const_ = 0.

**Figure 5:**
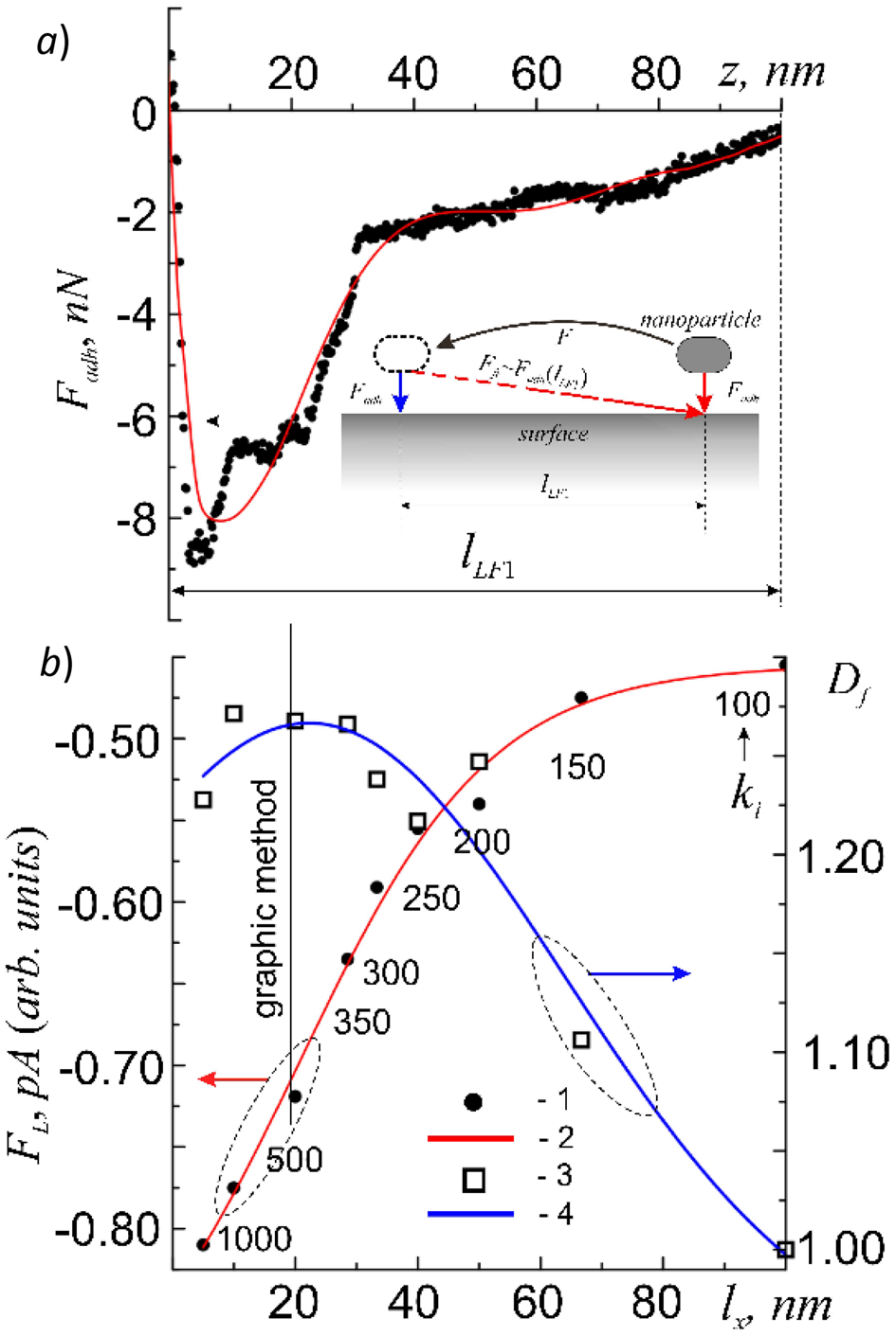
Dependence of the absolute values *F*_*adh*_ = *F*_*adh*_(*z*) for the probe-surface of the cell membrane system at F_const_ = 0 (points—experiment, line—approximation) (**a**), and relative averages over the 5 × 5 μm region of the values 〈*F*_*L*_(*l*_*x*_)〉, or 〈*F*_*adh*_(*k*_*i*_)〉 (points 1—experiment, line 2—approximation) and *D*_*f*_ = *D*_*f*_(*l*_*x*_) when moving the probe along the surface along *l*_*x*_ (points 3—experiment, line 4—approximation) (≈).

In repose, between the nanoparticle and the point or region of interaction with the surface (z = 0) located under it, adhesive Van-der-Waals forces of attraction arise and are described by the potential (3). The experimental dependence of these forces on the distance to the surface *F*_*adh*_ = *F*_*adh*_(*z*) is shown in Fig. 5a. It is important that the interaction of a particle (object) is carried out not with the entire surface, but with a limited area under the particle (object) approximately equal to the area of its projection, i.e. the cross section of the tip of the cantilever with a radius of < 6 nm. In such a case it can be assumed that the connection with the point of interaction (the place of landing) when the particle moves along the surface is also retained within *l*_*LF*1_* ≈ *100 nm. It follows from the electronic nature of chemical bonds that as long as one bond exists, a particle cannot form a second analogous bond with another place on the surface. When a particle moves along the surface from the place of initial interaction, the force *F*_*adh*_ = *F*_*adh*_(*x*) decreases according to the known law, and only at a distance *x* = *l*_*LF*1_* ≈ *100 nm, it becomes equal to zero. In order to break this connection, it is necessary to do some work A* ≈ *〈*F*_*adh*_〉*l*_*LF*_, which, in fact, will be equal to the work of friction forces. Only in this case the particle will be able to form a similar connection with a new local area on the membrane surface (Fig. 5a, inset). With further movement of the nano-object over the surface everything is repeated in the same way. As a result, we can assume that the movement of the nanoparticle over the membrane surface should be discrete. It is clear that such discreteness should manifest itself for objects whose sizes do not exceed or are commensurate with *l*_*LF*_. Objects that are much larger than *l*_*LF*_ will not experience noticeable discreteness in the friction force *F*_*fr*_ = *F*_*L*_ = *F*_*L*_(*x*) due to the fact that the resulting *F*_*L*_ value will always be averaged over the projection area (the contact area of the object). It should be noted that such discreteness is not associated with discreteness of the atomic structure of the surface.

**Figure 6:**
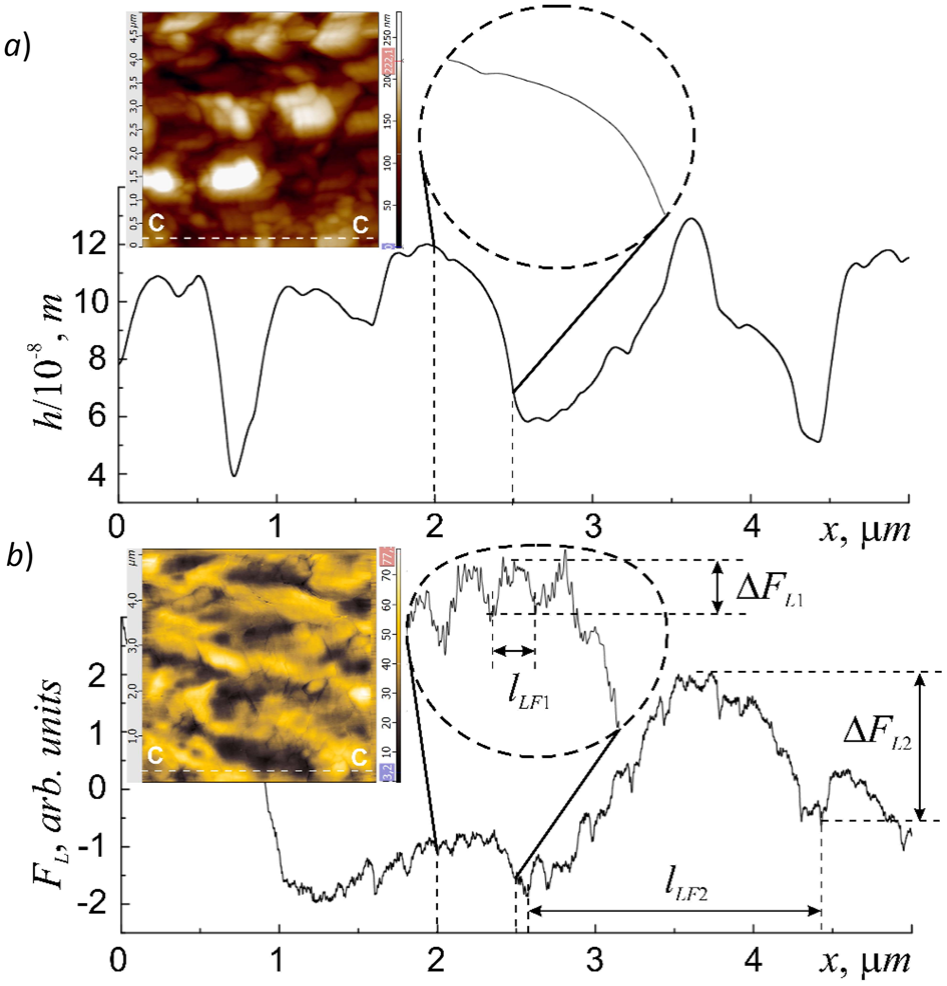
Profiles of cross-sections 5 × 5 μm of the surface area: relief h = h (x) (**a**), and LF-signal *F*_*L*_ = *F*_*L*_(x) (**b**). The insets show their bitmap images.

Direct measurements of *F*_*L*_ showed that on relatively smooth areas of the surface (Fig. 6a) the *F*_*L*_ value really changes abruptly due to discrete fluctuations Δ*F*_*L*1_ with an interval equal to *l*_*LF*1_* ≈ *100 nm (Fig. 6b). In this case the multifractal geometry of the membrane surface relief leads to the fact that the functional dependence *F*_*L*_ = *F*_*L*_(*x*) along the entire trajectory of the object's motion is also a multifractal curve. The average value of the fractal dimension, determined by the triangulation method shown in Fig. 6b of the *F*_*L*_ = *F*_*L*_(*x*) curve, was 〈*D*_*f*_ 〉= 1.24 and the surface relief *h* = *h*(*x*) – 〈*D*_*f*_ 〉= 1.29.

To study the behavior of the friction force depending on the size of micro- and nano-objects by the contact method in the LF method, a cyclic scanning of a 10 × 10 μm section with zero pressing force F_const_ = 0 was carried out with an increasing number of k_i_ scanning points in frame and line scans (i = 1, 2, …, 9; k_1_ = 100, k_2_ = 150, k_3_ = 200, k_4_ = 250, k_5_ = 300, k_6_ = 350, k_7_ = 500, k_8_ = 1000 and k_9_ = 2000). The absence of the pressing force makes it possible to exclude the deformation μ*F*_⊥_ in Eq. (2) and leave only the adhesive component F_adh_ of the friction force F_fr_ (13). This procedure makes it possible to study the effect of the adhesive component of the friction force, F_fr_, depending on the size of the measurement scale—the scanning step *l*_*i*_ = 10/*k*_*i*_: *l*_1_ = 100 nm, *l*_2_ = 66.7 nm, *l*_3_ = 50 nm, *l*_4_ = 40 nm, *l*_5_ = 33.3 nm, *l*_6_ = 28.6 nm, *l*_7_ = 20 nm, *l*_8_ = 10 nm and *l*_9_ = 5 nm. The measurement results were three-dimensional arrays of the values of the relief, h = h(x, y) and the lateral force *F*_*L*_ = *F*_*L*_(*x*,*y*) = *F*_*fr*_(*x*,*y*). Then, using the methods of mathematical statistics, from the distribution histograms *N*_*i*_ = *N*(*F*_*L*,*i*_) for each *k*_*i*_-th case the mean values 〈*F*_*L*,*i*_〉 and the variance *σ*_*i*_ were calculated. Recall that according to Section “Friction force” due to the impossibility of direct measurements of F_fr_ values it was necessary to operate with their relative values [*F*_*fr*_] = [*F*_*L*_] = p*A*. The result is shown in Fig. 5, b. It was found that an increase in the measuring scale–the scanning step *l*_*i*_, leads to a decrease in the modulus |*F*_*fr*_| keeping the values *σ*_*i*_ = 0.018–0.020. At *l*_*2*_ = 66.7 nm, the value of |*F*_*fr*_| goes to saturation.

In this case the fractality of the functional dependence *F*_*L*_ = *F*_*L*_(*x*) leads to the fact that with an increase in the size of the object from *l* to ≤ *l*_*LF*1_, the adhesion force and, consequently, the friction force in accordance with (11), decrease according to the power law (14):

14 $${\langle F}_{fr,i} \rangle =\langle {F}_{adh,i} \rangle =\langle {F}_{adh,0} \rangle {\left[\frac{{l}_{i}}{{l}_{0}}\right]}^{{D}_{T}-{D}_{f}}$$

This contradicts the nature of the manifestation of the friction force of macro-objects in the global approximation with *l* >> *l*_*LF*1_. For such macro-objects the friction force behaves in a classical way, i.e. it increases with an increase in their size and mass. Thus, it was observed that nanoparticles of large sizes in the local approximation under the condition *l* ≤ *l*_*LF*1_ can move along the surface of the cell membrane with a lower friction force.

Despite the absence of direct AFM methods for measuring the friction force *F*_*fr*_, the results obtained in this work still make it possible to find the absolute values of *F*_*fr*_. Let's use the graphical method. To do this it is necessary to align (compare) the scales along the ‘*z*’ and ‘*x*’ axes of the functional dependencies *F*_*adh*_ = *F*_*adh*_(z) (Fig. 5a) and *F*_*L*_ = *F*_*L*_(x) (Fig. 5b). When this is done one can easily determine which values of [*F*_*L*_] = pA in pico-Amperes on the *F*_*L*_ = *F*_*L*_(x) curve correspond to the values of [*F*_*adh*_] = n*N* in nano-Newtons on the *F*_*adh*_ = *F*_*adh*_(*z*) curve (Fig. 5, graphic method).

Thus, knowing the value of *D*_*f*_ of the cell membrane surface using Eqs. (13) and (14), one can easily determine the work of a particle that must be done to move it across the membrane surface.

According to the carried studies, an increase in the pressing force *F*_*const*_ in the area elastic deformations can lead to an increase in the effect of discretization of the friction force *F*_*fr*_, which manifests itself in an increase in discrete fluctuations Δ*F* and the sampling interval *l*_*LF*_. For example, an increase in *F*_*const*_ > 1 μ*N* leads to an increase in Δ*F*_*L*_ by a factor of ~ 2.5 and *l*_*LF*2_ almost to 1.5–2 μm as evidenced by the profile of the C–C section of the raster image of a periodically striped *LF-*signal on the membrane surface area of 5 × 5 μm (Fig. 6b) and 10 × 10 μm (Fig. 1b, inset). In the general case the value of the *l*_*LF*_ interval does not depend on either the resolution (the number of scan points), or on the speed, or on the scanning direction, but is determined only by the pressing force *F*_*const*_, which excludes the instrumental influence on the process of removing the *LF*-signal. Network structures with the manifestation of various types of scale bands are very characteristic of the structures of cell membranes (for example, the scale bands of 30 nm described in^33^).

Presumably, this tribological effect is determined by the fractality of the functional dependence *F*_*L*_ = *F*_*L*_(*x*). In the case when the level of external influence *F*_*const*_ begins to exceed the small fluctuations of one of the levels of statistical similarity *F*_*L1*_ and reaches or exceeds fluctuations of the next level of statistical similarity *F*_*L2*_ they begin to appear in the raster *LF* image *F*_*L*_ = *F*_*L*_(*x*,*y*) in the form of a more wide dark and light stripes with an increased period *l*_*LF*2_ (Fig. 6b, inset). This fact may indicate that micron-sized particles can also be influenced by the discrete nature of the friction force. It should be noted that the *LF-*contrast in the obtained raster images does not always coincide with the contrast of the relief as, for example, it was shown in^34^. This suggests that the forces of adhesion are not the main reason for the formation of the relief of the cell membrane and can manifest themselves regardless of its shape.

At the same time the axisymmetric structure of the *LF-*contrast relative to the cell nucleus does not allow for a complete lack of communication between the adhesive forces and the mechanical structure of the cell membrane formed by a network of glycan chains linked by peptide side branches, which in many cases has a local axisymmetric structure at the submicron and nanoscale levels (Fig. 1b) as, for example, the local regions of the membranes of gram-positive cells of the hay bacillus *Bacillus subtilis*, *Staphylococcus aureus*^28^, or *Lactococcus lactis* WT^34^.

The halo around the cell in the LF raster image, as indicated in Section “AFM methods”, is not associated with a violation of the membrane integrity but is formed by a precipitate from a buffer solution, the precipitation of which in the halo area was stimulated by the cell's own electrostatic field formed by the difference in electrostatic potentials Δφ between the cell and silicon surface (Fig. 2b). The electrostatic nature of the cell membrane requires a special study and was not considered in this work.

### Mapping the tribological properties of the buccal epithelium cell membrane

The study of the architecture of the outer shells of cells requires complex mapping of their functional characteristics with the necessary resolution both in absolute value and in spatial coordinates. As such functional characteristics (hereinafter referred to as characteristics), one can consider lateral forces *F*_*L*_ = *F*_*L*_(*x*,*y*) and adhesion forces *F*_*adh*_ = *F*_*adh*_(x, y). Due to the complex and time-consuming technical implementation of AFM measurement of the parameters of these characteristics, a several-point or grid method of local measurements with a very limited resolution of only 16 × 16 points (pixels) is often used as, for example, in^34^, aimed mainly at increasing the statistical sample. In this case, the sizes of local areas usually do not exceed a few micrometers, which makes it difficult to study the architecture of the external exoskeleton of the cell membrane as well as the relationship of the morphology of the membrane surface with its micromechanical and tribological characteristics. Currently there are no general recommendations for choosing the necessary scanning conditions. Everything is determined by the capabilities of the AFM, the requirements of the researcher, the size and condition of the cell, and external conditions.

The cells of the human buccal epithelium belong to rather large biological micro-objects–the size of adult eukaryotes can reach hundreds of micrometers, with a sufficiently developed landscape of the surface of their membranes. The mapping of the complex functional characteristics of such objects, in particular, the adhesion forces and lateral forces, was carried out in 10 × 10 µm regions with a minimum resolution of 100 × 100 scanning points in frame and line scans.

To solve the problem of mapping by AFM methods a hybrid mode was used, which made it possible to simultaneously obtain data on the relief and the above tribological surface properties at each scanning point. With the aim of reproducibly obtaining and registering at each point of the measurement results, i.e. the approach and withdrawal curves, an extended range of external force effects from 0 to 40 µN was used with a sufficiently high level of the initial force action *F*_*const*_ ≈ 21 µ*N* significantly exceeding the level required for the appearance of elastic deformations Δ*h*_*dfrm*_. The case of destructive deformations was not considered in this work.

**Figure 7:**
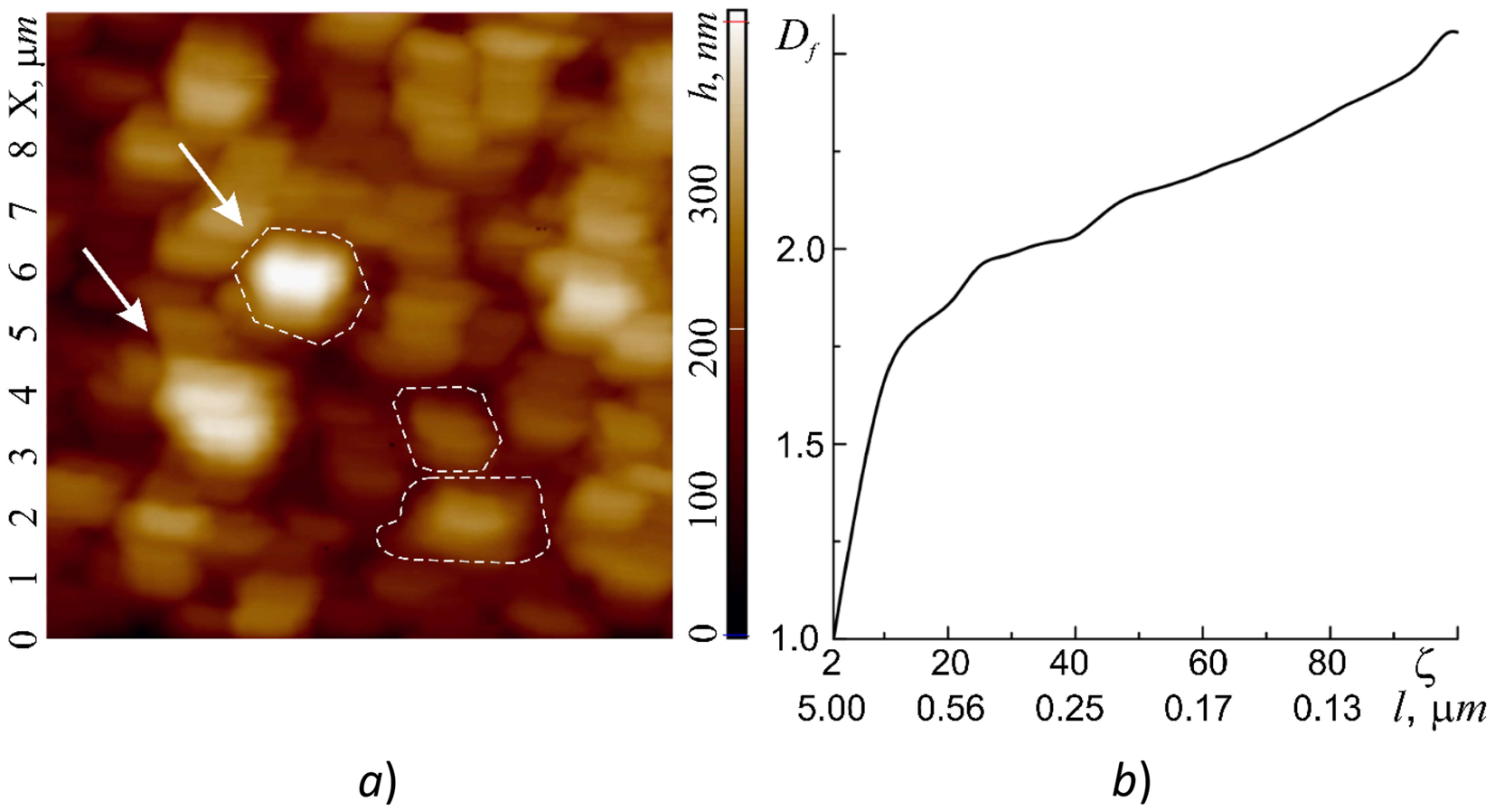
Image of a 10 × 10 μm region of the cell wall surface obtained in the hybrid AFM mode at a resolution of 100 × 100 scanning points (some elements of the cellular structure are circled with a dotted line): relief h = h(x, y) (**a**) and dependence of fractal dimension *D*_*f*_ = *D*_*f*_(*ζ*) on the values of the scaling coefficient *ζ* (**b**). Under the values of *ζ* the corresponding for the investigated area values of the measuring scales *l* are shown.

As reported in Section “Tribological properties of the cell membrane surface”, the outer shell of adult living cells of human buccal epithelium is not a smooth surface, but has a self-affine multifractal Brownian relief with an average vertical size of irregularities (tubercles) up to 500 nm. In the horizontal plane the surface has a rather coarse cellular structure ranging in size from 200 to 2000 nm (Fig. 7a, highlighted by a dotted line). The size of the irregularities depends on the age of the cell and its condition. More detailed studies of the cells (in the semicontact scanning AFM mode at a higher resolution of 500–500 points), in turn, revealed their grid structure with sizes from 50 to 350 nm (Fig. 7). It should be noted that the mesh structure of the exoskeleton at the nanoscale is very widespread among cell membranes (for example^28^). In this case it clearly manifests its fractal geometry not only at submicron level, but also at micron level. Of interest are the size and shape of such cells, which, according to the available data, can be ‘bags’ of a disordered porous peptidoglycan gel—a three-dimensional network in a liquid medium^35^.

In the previous section it was shown that the tribological properties of cell membranes at nanoscale level are almost completely determined by the action of adhesive forces. In this regard it is of interest to map the adhesion forces *F*_*adh*_ = *F*_*adh*_(*x*,*y*), and friction forces *F*_*fr*_ = *F*_*L*_, which are essentially equal to the lateral forces *F*_*L*_ = *F*_*L*_(*x*,*y*) ≡ *LF*(*x*,*y*) (*LF*-contrast). From the contrast of the raster image *F*_*adh*_ = *F*_*adh*_(*x*,*y*) it is also possible to determine the shape, size of cells and their boundaries, the position of which fully corresponds to the raster images of the shell relief section (Fig. 7a). The range of *F*_*adh*_ variation at an increased level of external influence can vary in a fairly wide range from 0 to ~ 45 µN. It is clearly seen that the local sections of cells with different elastic and plastic properties can have different adhesive forces. The highest values of *F*_*adh*_ are reached at the boundaries of the cells and the lowest—at the areas with the lowest plastic deformations and the highest elastic properties. The average value of the fractal dimension *F*_*adh*_ = *F*_*adh*_(*x*,*y*) determined by the triangulation method was 〈*D*_*f*_(*F*_*adh*_)〉 = 2.78. The histogram *N* = *N*(*F*_*adh*_) is well described by three Gaussian functions.

**Figure 8:**
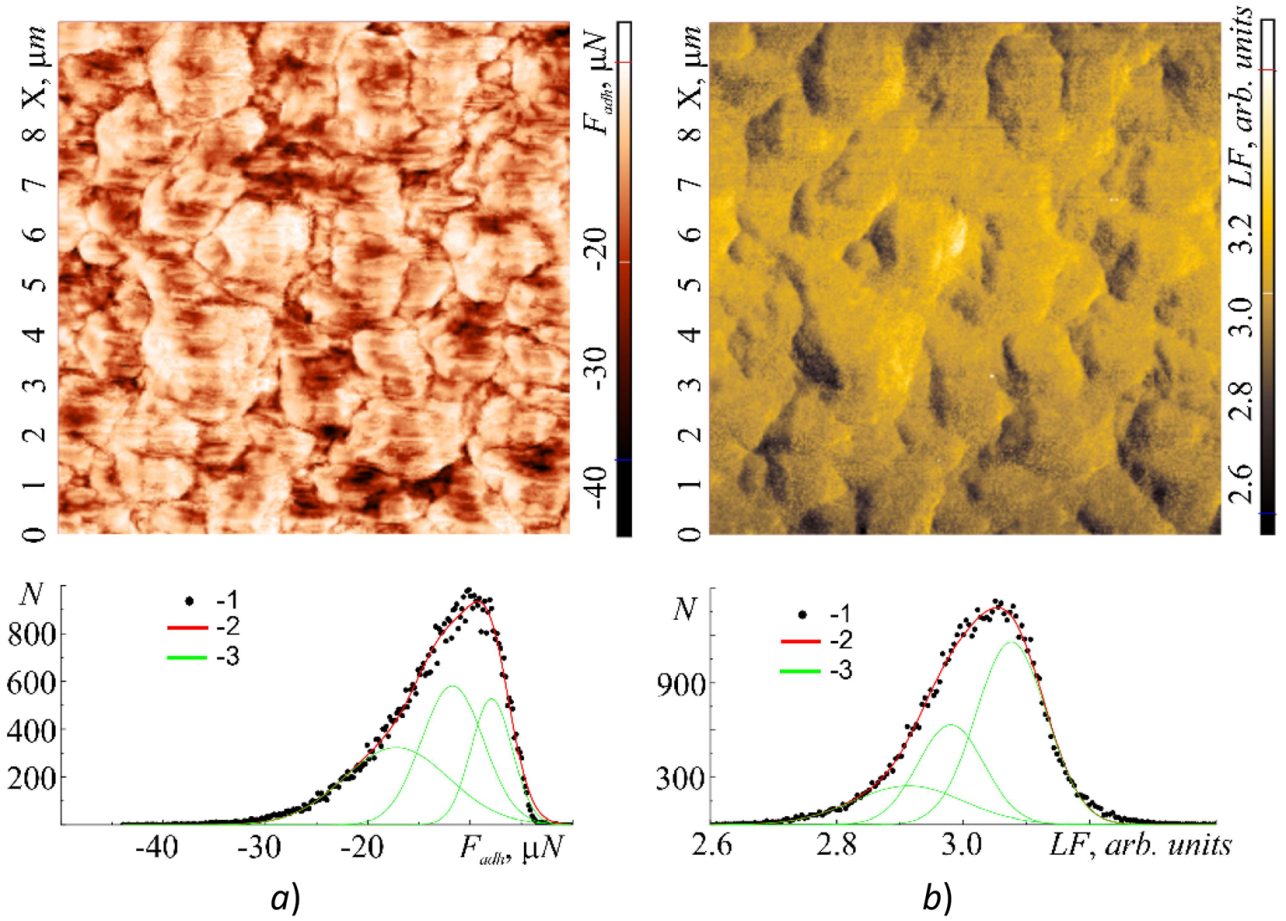
Topographic images of a 10 × 10 μm section of the cell wall surface obtained in the hybrid AFM mode at a resolution of 100 × 100 scanning points: adhesive *F*_*adh*_ = *F*_*adh*_(*x*,*y*) (**a**) and lateral *F*_*L*_ = *F*_*L*_(*x*,*y*) (*LF*-contrast) (**b**) with their histograms *N* = *N*(*F*_*adh*_) and *N* = *N*(*LF* ≡ *F*_*L*_)—(2) respectively. Points (1)—experiment, curves (3)—approximation by Gaussian functions.

Comparing Fig. 8a and b one can see that the maximum modulus values of adhesive forces |*F*_*adh*_| correspond to the maximum modulus values of lateral forces |*F*_*L*_| and, accordingly, friction forces |*F*_*fr*_|. The histogram *N* = *N*(*F*_*L*_) is also well described by three Gaussian functions, which correlates with the number of Gaussians in *N* = *N*(*F*_*adh*_). This confirms the important role played by the adhesion forces in the formation of friction forces at the nanoscale level. The average value of the fractal dimension *F*_*L*_ = *F*_*L*_(*x*,*y*) determined by the triangulation method was 〈*D*_*f*_(*F*_*L*_) 〉= 2.80, which practically coincides with the average value 〈*D*_*f*_(*F*_*adh*_)〉 = 2.78.

## Conclusion

In this work the morphology and tribological properties of the membrane of living cells of human buccal epithelium in the presence of a protective adsorption layer of ~ 100 nm buffer solution were studied using atomic force microscopy in the contact scanning mode.

The outer shell of adult living cells of human buccal epithelium is not a smooth surface, but has a self-affine multifractal Brownian relief with an average vertical size of irregularities (tubercles) up to 500 nm. In the horizontal plane the surface has a rather coarse sectional structure with sizes from 200 nm to 2 µm. The sizes of sections and irregularities depend on the age of the cell and its state. More detailed studies of the cells, in turn, revealed their grid structure with sizes from 50 to 350 nm. Of interest are the size and shape of such cells, which, according to the available data, can represent ‘bags’ of a disordered porous peptidoglycan gel—a three-dimensional network in a liquid medium.

In addition to the shape of the relief the determining value in the transport functions of the cell membrane of the buccal epithelium cell along its surface is the friction force *F*_*fr*_, which, as shown in the work, is completely determined by the adhesion Van-der-Waals forces. In a local approximation using the mathematical apparatus of fractal geometry, the dimensional effects of the process of friction of micro- and nanoobjects on the surface of the cell membrane are described. The fractal geometry of the surface relief of the cell membrane leads to the fact that the functional dependence of the friction force *F*_*fr*_ = *F*_*fr*_(*x*) on the entire trajectory of the object is a fractal line. In this case the fractality of the functional dependence of the friction force on the coordinate *F*_*fr*_ = *F*_*fr*_(*x*) leads to the fact that with an increase in the size of the object from *l* to ≤ *l*_*LF,*_ its absolute value decreases according to a power law, which is in a contradiction with a similar dependence of the friction force in the global approximation for macroobjects. Direct measurements of *F*_*fr*_ on the membrane surface showed that in relatively smooth areas its value changes abruptly with an interval equal to *l*_*LF*_ ≈ 100 nm. It was shown that such discreteness will manifest itself for objects whose dimensions do not exceed or commensurate with the *l*_*LF*_ interval of the action of the adhesion forces *F*_*adh*_. Objects that are much larger than *l*_*LF*_ will not experience noticeable discreteness in *F*_*fr*_ = *F*_*fr*_(*x*) due to the fact that the resulting *F*_*fr*_ value will always be averaged over the contact area.

In this work during tribological studies in the contact mode of AFM scanning a unique method was developed for determining the absolute value of the friction force of nanoobjects on the surface of the cell membrane. As a result the absolute value of the friction force of the tip of the cantilever needle against the surface of the cell membrane was measured for the first time in the contact AFM scanning method with a constant pressing force. This method is universal and can be used to determine the absolute values of friction forces between objects of inanimate nature.

In a local approximation the tribological parameters of the cell membrane surface [friction forces *F*_*L*_ = *F*_*L*_(*x*,*y*) and adhesion forces *F*_*adh*_ = *F*_*adh*_(*x*,*y*)] were mapped.

The values of the parameters obtained in the work depending on the state of the cell, may differ in one direction or another. Further research is needed to answer this question.
